# The Averaging Effect of Odorant Mixing as Determined by Air Dilution Sensory Tests: A Case Study on Reduced Sulfur Compounds

**DOI:** 10.3390/s110201405

**Published:** 2011-01-26

**Authors:** Ki-Hyun Kim

**Affiliations:** Atmospheric Environment Laboratory, Department of Environment & Energy, Sejong University, Seoul 143-747, Korea; E-Mail: khkim@sejong.ac.kr; Tel.: +82-2-3408-3233; Fax: +82-2-3408-4320

**Keywords:** human sensing, odor mixing, threshold, hydrogen sulfide, methanethiol, dilution-to-threshold (D/T) ratio

## Abstract

To learn more about the effects of mixing different odorants, a series of air dilution sensory (ADS) tests were conducted using four reduced sulfur compounds [RSC: hydrogen sulfide (H_2_S), methanethiol (CH_3_SH), dimethylsulfide (DMS), and dimethyldisulfide (DMDS)] at varying concentration levels. The tests were initially conducted by analyzing samples containing single individual RSCs at a wide range of concentrations. The resulting data were then evaluated to define the empirical relationship for each RSC between the dilution-to-threshold (D/T) ratio and odor intensity (OI) scaling. Based on the relationships defined for each individual RSC, the D/T ratios were estimated for a synthetic mixture of four RSCs. The effect of mixing was then examined by assessing the relative contribution of each RSC to those estimates with the aid of the actually measured D/T values. This stepwise test confirmed that the odor intensity of the synthetic mixture is not governed by the common theoretical basis (e.g., rule of additivity, synergism, or a stronger component model) but is best represented by the averaged contribution of all RSC components. The overall results of this study thus suggest that the mixing phenomenon between odorants with similar chemical properties (like RSC family) can be characterized by the averaging effect of all participants.

## 1. Introduction

In processing olfactory information, the mixing of individual odorants is often treated as one of the most puzzling areas of study due to either the complexities involved in the identification of the resulting product or to the present limits in our knowledge to offer a quantitative description of such a phenomenon. The results of previous studies confirm that human olfactory perception decreases dramatically in terms of the identification capacity with the complication of the mixture. For instance, Laing and Francis [1] demonstrated that the identification success rate dropped exponentially with the increasing number of ingredients, e.g., for mono (55%), binary (12%), ternary (6%), and quaternary mixtures (3%).

The identification capacity is an important component of the mixing process, as is the strength of the produced odorant mixture. One of the simplified answers to address as the rules of olfactory detection may be the linear additive trend [2]. However, it is not necessarily that simple in practice, as such effects can come out in all possible directions: (1) masking or dominance by a stronger component (e.g., [3]), (2) hypoadditivity (lower than the sum or average) [4,5], and (3) synergistic effects [6,7]. Because of the complexities involved in these unstoichiometric phenomena, it is not easy to describe or demonstrate the outcome or effects of mixing in a predictable and systematic manner. There are only a few limited tools or techniques to describe the relationships between concentration-detection functions of mixing. For instance, Cometto-Muniz *et al.* [5,8] approached this problem by first acquiring psychometric functions of individual odorants and then by attempting to detect their mixtures of varying composition. Based on this approach, these authors were able to find that the patterns of olfactory detection or the completeness of dose addition varies greatly with the magnitude of detectability set for the individual ingredients of the mixture.

As one possible option for defining the dose addition relationship of odorants, we introduced a new testing approach in which relationships established between single odorants are used for those of mixtures by means of the dilution-to-threshold (D/T) ratios derived by the air dilution sensory (ADS) test [9]. Based on this approach, we were able to show that the competing relationships hold between different odorants contained in the mixture of H_2_S and many carbonyls and that the patterns of masking phenomena change with their concentrations. In order to continue our efforts to investigate the odor mixing phenomenon, our testing approach for the dose addition relationship was employed to investigate the mixture of four reduced sulfur compounds (RSCs). Based on these experiments, we discuss how the effect of mixing occurs between the odorants of the same family (or similar chemical properties). The results of our initial research on the effects of mixing between different odorant families (*i.e.*, RSCs and aldehydes) have been reported elsewhere [3].

## 2. Materials and Methods

### 2.1. Experimental Scheme for Masking Effects

In this study, the effects of synthetic mixing were investigated using four individual RSCs consisting of hydrogen sulfide (H_2_S), methanethiol (CH_3_SH), dimethylsulfide (DMS), and dimethyldisulfide (DMDS). These compounds were selected because of their significance as the key offensive odorant group designated by the malodor prevention law of Korea [10]. The environmental impact of RSC in terms of malodor components released by industrial activities has been described in a number of previous studies [11–13].

**Figure 1.**
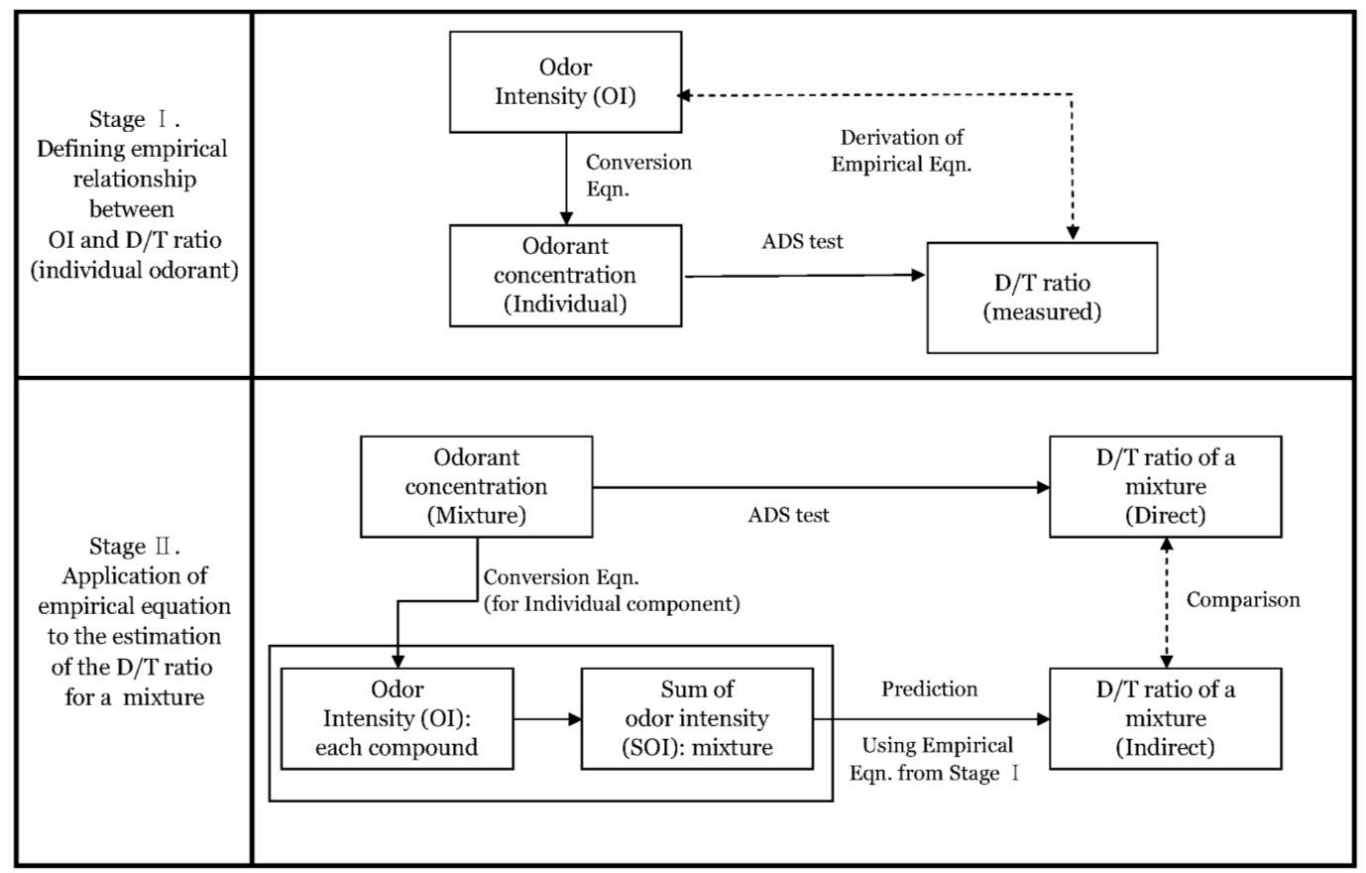
Schematic of the two-stage approaches for the comparison of D/T ratios between measured and predicted values.

The basic experimental scheme of our study is presented in Figure 1. The major components of this study can be divided into two stages. In the first stage, the relationships between different expression units for odor composition [e.g., odor concentration *vs.* odor intensity (OI)] are defined based on the pre-existing definition of their interactions [14,15]. More specifically, OI is first calculated from the known concentrations of each RSC whose samples are prepared to represent 11 OI ratings (Table 1). These individual samples are then subject to the ADS test to assign the corresponding D/T ratios to each individual sample. After all, by binding all these concepts (concentration, OI, and D/T ratios), one can eventually establish empirical relationships between the odorant concentration and the D/T ratios, which were not initially linked. At the next stage, the newly established equations from the stage 1 experiment is then used to estimate the D/T ratios of the four RSC mixture (M_4_) samples in relation to each individual component. Hence, information concerning these estimated D/T results in stage 1 is used to assess the contributions of individual RSC components to the overall strengths of odor mixture in stage 2.

### 2.2. The Preparation of Malodor Samples

In the course of our study, RSC samples for the ADS test were prepared based on two different criteria. In the first stage of the ADS experiment, samples for four individual RSCs were prepared by using a permeation tube (PT) device for their respective standard gases (Metronics, CA, USA). All of these PT-based samples were cross-calibrated by a cylindered-based standard containing four RSCs at near-equimolar concentration levels of 10 ppm (Ri gas, Korea). In order to define empirical relationships between the concentration levels (or OI) of individual RSCs and their D/T ratios, samples were prepared to match a wide range of odor intensities (*i.e.*, 11 levels in this study) that end in one decimal point with either 0 or 2 such as 0.2, 1.0, 1.2, 2.0, and so on (Table 1).

To comply with our experimental scheme in the second stage, the odor mixture samples containing four RSCs at the same time (M_4_) were prepared at 11 concentration levels using the above described cylinder standard (Table 2). Note that RSCs of the same concentration levels can exhibit a relatively broad range of the OI ratings, as their odor threshold values differ greatly (Table 2). As such, the 11^th^ standard of the M_4_ sample (∼1,000 ppb for all 4 RSCs) shows OI values in the range of 4.06 (DMS) to 6.02 (CH_3_SH) which approximately correspond to the difference of two orders of magnitude in their odor strengths. This distinction in OI ratings between CH_3_SH and other RSCs in the standard gas mixture is thus useful to distinguish the effects of mixing on the overall odor strengths acquired. Because of this superior threshold of CH_3_SH in all the M_4_ mixture compositions, it generally dominates the estimated D/T levels throughout all 11 samples prepared at varying concentrations (Table 2). Overall, this pronounced pattern of mixture composition (*i.e.*, predominance of CH_3_SH in terms of OI) indeed helped us evaluate the actual destination of odor mixing processes between a variety of competing odorants.

### 2.3. Air Dilution Sensory (ADS) Test Based on Olfactometry Threshold Method

In this work, the actual application of the air dilution sensory (ADS) test is made by the standard procedure established by the Korean Ministry of the Environment [9]. As described in our companion paper [3], the KMOE method of the ADS test belongs to a threshold olfactometry in which the central trend of the odor index value is derived geometrically for a given odor sample, after excluding the data sets of the two extreme ends obtained from each round of testing. The samples prepared either individually or as mixtures were then subject to the ADS test by a panel of five members; all of these members were selected based on a pre-screening test in which all participants are requested to differentiate samples of deionized water from testing solutions containing four chemicals with the following weights (%) of acetic acid (1), TMA (0.1), methylcyclopentenolone (3.2), and β-penylethylalcohol (1).

The static dilution of odorant samples for the ADS test was made in a stepwise manner by mixing the original odorant samples with odorless air using a 3 L odor bag made of polyethylene telephtalate film. The odorless air used was prepared by passing normal air into an activated charcoal filter. The ADS test was conducted continuously using odorant samples prepared through a stepwise dilution. This test was conducted until the last panel member reached the minimum detection (threshold values) of a given odor sample. The level of dilution for the ADS test progressed through an application of the multiplying factors derived as X values:

$$X=Z\;10^{n}$$

Here, the superscripted value ‘n’ corresponds to an integer value of 0, 1, 2, 3,…., n. In addition, Z is a multiplying factor of either 1 or 3. The odor index value for a given sample is then processed by the stipulated method of KMOE [9]. The results of the 2-stage ADS experiments are ultimately expressed as D/T ratios through a combination of the ‘yes/no’ opinions from all panel members.

## 3. Results and Discussion

### 3.1. Concentrations of Individual RSCs vs. Their Corresponding D/T Ratios

**Figure 2.**
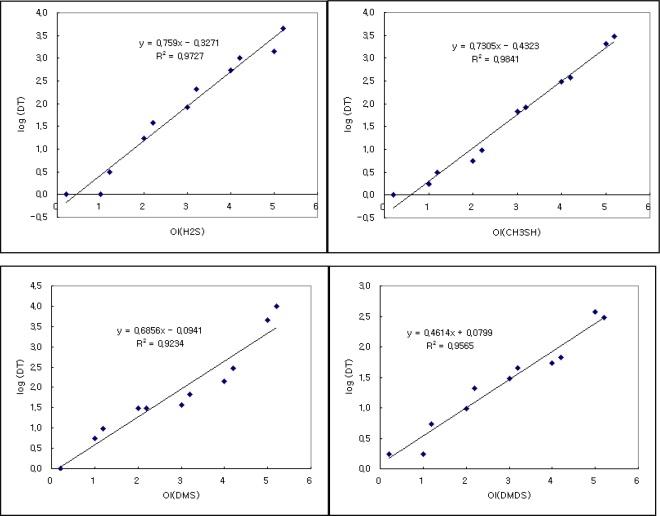
Relationship between odor intensity [by equations in Table 2(A) and the measured D/T ratio of 4 reduced sulfur compounds (RSCs)].

A schematic of our data evaluation is depicted in Figure 1. In the first stage of our analysis, the results of individually prepared RSC samples are used to derive relationship between concentration levels and the corresponding D/T ratios. To this end, the concentrations of each individual RSC are first converted to their corresponding OI ratings. The results of the ADS tests for each individual RSC, expressed in terms of D/T ratios, are presented in Table 3 for a total of 11 samples. These OI values are plotted against the D/T ratios measured from the ADS test to derive empirical equations between converted OI values and D/T ratios (Figure 2). In the case of the H_2_S, the sample with the lowest OI value of 0.2 (or 0.07 ppb in concentration) yielded a D/T ratio of 1, while the sample with the maximum OI of 5.2 recorded a D/T ratio of 4,481. Hence, the concentration ratio of H_2_S between the maximum and minimum (13,055/0.07 = 1.9 × 10^5^) is greatly distinguishable from its D/T counterpart (4,481/1 = 4.5 × 10^3^). If this comparison is extended to CH_3_SH, these ratio values are computed at much reduced levels. For instance, their corresponding ratio values are reduced by more than an order of magnitude to 1.2 × 10^4^ (=233/0.02) and 3 × 10^3^ (=3,000/1), respectively. It should be addressed that the slope value of H_2_S observed in this study (0.76) is slightly reduced relative to the one we introduced in our initial study (slope value of 0.78) conducted with the mixture of H_2_S and aldehydes [3].

According to this comparison, the relative ordering of human perception between different RSCs can be distinguished fairly systematically. It confirms that the slope values of four RSCs decrease gradually and systematically with increasing molecular weights. For instance, the lightest molecule, H_2_S exhibits the largest slope value of 0.76 with a small negative offset value. In contrast, the heaviest (DMDS) has the least slope value of 0.46. Hence, changes in the D/T ratio tend to proceed very rapidly for H_2_S, while the heaviest DMDS goes most slowly across the entire OI range. In other words, human perception can occur more actively in the lighter RSCs rather than the heavier counterparts. It is interesting to note that there is a slight reduction in slope value of CH_3_SH (0.73) relative to H_2_S. This observed pattern between the two RSCs thus suggests that the threshold detection property of RSC can be greatly distinguished from their responsive relationships between OI and D/T ratios.

### 3.2. Estimation of D/T Ratios for RSC Mixture

As explained above, the relationship between RSC concentrations and all the related parameters (e.g., odor intensity and D/T ratio) can be basically defined for any individual RSC. In contrast, the evaluation of odor intensity for an RSC mixture becomes a more complicated task than that for a particular odorant. In order to simplify the assessment of the OI levels for RSC mixtures (in this study, the M_4_ samples), the sum of odor intensity (SOI) concept was applied to each mixture sample by binding the OI values of individual RSCs in a logarithmic scale [8] as follows:

$${\text{SOI}}_{i}=\text{log}\;(10^{\text{OI(i)}1}+10^{\text{OI(i)}2}+10^{\text{OI(i)}3}+\cdot \cdot \cdot \cdot \cdot \cdot \cdot \cdot \cdot \cdot \cdot \cdot \cdot \cdot +10^{\text{OI(i)n}})$$

where OI(i) = the odor intensity of an individual RSC in the “i”th stage standard mixture. The subscript numbers 1 through n correspond to the order of the individual components of the mixture. According to this conversion formula, the first M_4_ sample consisting of ∼0.10 ppb concentration levels is computed to have an SOI value of 1.40 (Table 4). Likewise, the last M_4_ sample has a corresponding SOI value of 6.04, as shown in Table 4. Because SOI values can be assigned to any kind of mixture with a complicated composition, comparison of odor strengths for the M_4_ sample in this study can be made on a parallel basis.

**Figure 3.**
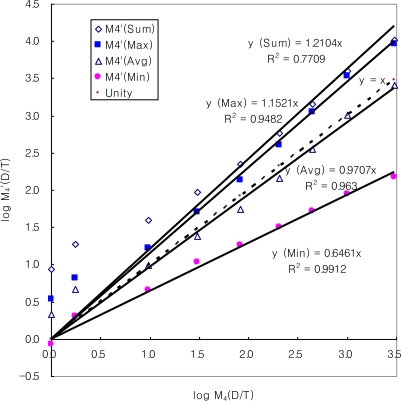
Correlation analysis of log (D/T) values between measured values of log (M_4_) and 4 types of statistical derivatives with prime symbol (M_4_’).

In order to estimate the relative contribution of individual RSCs to the strength of its mixture, the D/T ratios for mixture samples have been evaluated between measured and predicted values in a stepwise manner. It should first be noted that the D/T ratios for a single odorant can be calculated for the samples with any OI values through equations, as defined by the empirical relationships (Figure 2). This type of approach used for individual RSCs can now be extended further to predict the D/T ratios for the RSC mixture. To initiate this estimation, an RSC mixture sample with a given SOI value is assumed to be represented by any single constituent that has the equivalent OI level. The first M_4_ sample has an SOI value of 1.40 with a measured D/T ratio of 10^0^. The D/T ratios for this mixture sample can thus be first approximated by the single odorant with the same odor intensities. Under such an assumption, the odor intensity scaling of this mixture is estimated by accounting for the contribution of each single ingredient (H_2_S or three others) at each concentration level. For instance, an RSC mixture of the lowest concentration level has SOI (or OI) values of 1.40 (Table 4). The D/T ratios of its individual constituents are then estimated to fall in a wide range of 10^−0.07^ (H_2_S) to 10^0.54^ (DMS), as shown in Table 2(C). Because each single component consisting of the synthetic mixture samples can make different contributions, the D/T values estimated for each individual RSC component can later be used to assess their relative roles through a direct comparison with the measured D/T ratios (Figure 3).

### 3.3. Comparison of estimated *vs.* measured D/T ratios

As shown in Table 4, the D/T ratios of mixed odorants can be roughly estimated by the alternate single component through the empirical relationship and can be compared with the measured counterparts in a diverse manner. Because D/T ratios of the mixture, M_4_, are initially estimated from the four individual RSCs, the actual measurement for their mixture, M_4_ can be compared with their statistical derivatives (like maximum, minimum, sum, and average) of all four components (H_2_S, CH_3_SH, DMS, and DMDS).

To learn more about the interactive roles between different components in the mixture, the evaluation of the M_4_ mixture data can be extended further by considering their relationships with those of diverse derivatives extracted from the four individual RSCs (Table 4). In Figure 3, the measured log D/T ratios for the RSC mixture (M_4_) are thus compared with four types of the predicted log D/T values with prime symbol; here, the contribution of individual components to the mixture is estimated in terms of simple numerical combinations such as average, sum, maximum, and minimum. Specific procedures used for the estimation of log D/T values are explained in the footnotes of Table 4. According to this comparative approach, two contrasting patterns emerge. In the lower log D/T range below 0.98, two data points exhibit relatively large gaps between measured and predicted values. Because the measured D/T values are much lower than the max, sum, and average, some kind of masking effect can occur at this lower D/T range. However, once the data obtained at this delicate range are excluded, the results are consistent enough to show a constant trend in which the averaged D/T ratios with the slope values approaching the unity (0.97) copy the behavior of mixture determined by direct measurements (dotted line in Figure 3).

In the sense that average is smaller than sum but larger than minimum, this phenomenon can still reflect some kind of masking. However, this observed averaging effect is greatly distinguished from the common masking in which the maximum component overwhelmingly represents all ingredients. More specifically, our observation of the best compatibility for a RSC mixture with the averaged D/T components differs greatly from the results we obtained from a mixture of two different chemical groups between H_2_S and aldehydes [3]. In our previous study, when those two groups were put together to produce M_2_ (H_2_S and acetaldehyde) and M_6_ mixtrues (H_2_S and five aldehydes), the measured D/T ratios were represented most efficiently by the maximum D/T components derived from the competing relationship between H_2_S and one of the aldehydes (e.g., acetaldehyde in M_2_ and isovaleraldhyde in M_6_) among all participating constituents at a given OI range [3]. Thus, the results of our previous experiments between two different chemical groups showed a good agreement with the general definition of masking or dominance by a stronger component (e.g., [3]). However, the results of the present study in which H_2_S was mixed with three other components of the same family group can show an averaging pattern that is entirely different from the ones derived when added with other chemical groups (aldehydes). Although mixing of odorants is known to yield diverse patterns of mixing phenomena (e.g., masking, hypoadditivity, and synergistic effect), the results obtained from this study stress the importance of the “average effect” as one option for the outcome of the odorant mixing process.

## 4. Conclusion

In this research, the basic aspects of synthetic mixing between odorants of the same chemical groups were tested using standard gases of four reduced sulfur compounds prepared to represent 11 concentration levels. The relationships between their concentrations and the odor intensities (OI) were tested using their standards of both the four individual components and their mixture through the air dilution sensory (ADS) test in terms of the dilution-to-threshold (D/T) ratio. The relationship established from the individual components (stage 1) was used to estimate their contributions to the mixture of 4 RSCs (M_4_) prepared across 11 concentration levels (stage 2). The effect of synthetic mixing was then evaluated by considering the relationship between the estimated and measured D/T ratios of the mixture. The results of our comparative analysis of the D/T ratios between estimation and direct measurement indicate that the odor intensity of a RSC mixture is determined by the average effect of all individual RSC components rather than other common styles of the mixing effect (e.g., masking, hypoadditivity, or synergistic effect). Overall, the direct quantification of D/T ratios by the human test panel was helpful in demonstrating the occurrence of the averaging phenomenon between odorants of similar chemical properties. The pattern of odorant mixing observed from the same chemical groups (between H_2_S and three other RSCs) in the present study is thus clearly distinguished from the previously-observed masking phenomena (between H_2_S and aldehydes) that had been based on the same experimental approach. The overall results of this study confirm that the effect of odorant mixing can be expressed in highly diversified directions and that chemical similarity can be a key parameter determining such directions.

## Figures and Tables

**Table 1.** Preparation of RSC standard for air dilution sensory (ADS) test.

**A. Relationship between OI and concentration (ppm)^a^: OI = a log C + b**					

Order	Parameter	H_2_S	CH_3_SH	DMS	DMDS

1	Slope (a)	0.95	1.25	0.784	0.985
2	Offset (b)	4.14	5.99	4.06	4.51

**B. Computation of RSC standard concentrations (ppb) for selected OI values**					

Order	Odor intensity	Concentration (ppb)^b^			

		H_2_S	CH_3_SH	DMS	DMDS

1	0.2	0.07	0.02	0.01	0.04
2	1.0	0.50	0.10	0.13	0.27
3	1.2	0.80	0.15	0.22	0.44
4	2.0	5.59	0.64	2.36	2.83
5	2.2	9.08	0.93	4.24	4.52
6	3.0	63.1	4.06	44.5	29.3
7	3.2	102	5.86	80.0	46.8
8	4.0	712	25.6	838	304
9	4.2	1,157	37.0	1,509	484
10	5.0	8,040	161	15,812	3,144
11	5.2	13,055	233	28,450	5,018

a Nagata [14,15].

b Concentration of RSC standard for the selected odor intensity (OI) is calculated by combining the parameters and the formula given above.

**Table 2.** Preparation of odorant mixture consisting of 4 RSCs (M_4_) and the relationship between odor intensity and dilution-to-threshold (D/T) ratio.

**A. Detailed information of individual odorants added for a mixture odorant of M_4_**					

Order	Individual compound				

	H_2_S	CH_3_SH	DMS	DMDS	
	
A. Concentrations of odorants used for the mixed standards (ppb)					
1	0.10	0.11	0.10	0.11	
2	0.33	0.35	0.34	0.35	
3	1.00	1.05	1.01	1.05	
4	3.33	3.50	3.37	3.50	
5	10.0	10.5	10.1	10.5	
6	33.3	35.0	33.7	35.0	
7	100	105	101	105	
8	333	350	337	350	
9	1,000	1,050	1,010	1,050	

**B. Odor intensity (OI) of the above-listed individual odorants added for M_4_^a^**					

1	0.34	1.02	0.93	0.59	
2	0.84	1.67	1.34	1.11	
3	1.29	2.27	1.71	1.58	
4	1.79	2.92	2.12	2.09	
5	2.24	3.52	2.50	2.56	
6	2.74	4.17	2.91	3.08	
7	3.19	4.77	3.28	3.55	
8	3.69	5.42	3.69	4.06	
9	4.14	6.02	4.06	4.53	

**C. Estimation of log (D/T) for individual odorants by its relationship with OI^b^**					

1	(0.07)	0.31	0.54	0.35	
2	0.31	0.79	0.82	0.59	
3	0.65	1.22	1.08	0.81	
4	1.03	1.70	1.36	1.04	
5	1.37	2.14	1.62	1.26	
6	1.75	2.61	1.90	1.50	
7	2.09	3.05	2.15	1.72	
8	2.47	3.53	2.44	1.95	
9	2.82	3.96	2.69	2.17	

Superscripts a and b denote the relationship explained in Tables 1(A) and 2(B), respectively.

**Table 3.** Derivation of empirical relationship between OI and D/T ratio for each RSC.

**A. Results of air dilution sensory (ADS) test for each RSC at 11 OI levels**					

Order	Odor intensity	Dilution-to-threshold (D/T) ratio			

		H_2_S	CH_3_SH	DMS	DMDS

1	0.2	1.0	1.0	1.0	1.8
2	1.0	1.0	1.8	5.5	1.8
3	1.2	3.1	3.1	9.7	5.5
4	2.0	17.0	5.5	30.0	9.7
5	2.2	38.0	9.7	30.0	20.8
6	3.0	81.8	66.9	36.7	30.0
7	3.2	208	81.8	66.9	44.8
8	4.0	548	300	144	54.8
9	4.2	1,000	367	300	66.9
10	5.0	1,442	2,080	4,481	373
11	5.2	4,481	3,000	10,000	300

**B. Relationship between log D/T ratio and OI values for each RSC^a^**					

Order		H_2_S	CH_3_SH	DMS	DMDS

1	slope	0.759	0.7305	0.6856	0.4614
2	offset	−0.3271	−0.4323	−0.0941	0.0799
3	r^2^	0.9727	0.9841	0.9234	0.9565

a The data shown in the section (A) are used to derive the following equation, log (D/T) = a*OI + b based on linear regression analysis. Here a and b correspond to slope and offset, respectively.

**Table 4.** Comparison of various log (D/T) ratios between measured (M_4_) and 4 types of its predicted values (M_4_’) at each of all corresponding SOI values.

**Order**	**SOI**	**ADS result**	**Predicted values^a^**			
		**M_4_(M)**	**M_4_’(E-Max)^b^**	**M_4_’(E-Min)^c^**	**M_4_’(E-Sum)^d^**	**M_4_’(E-Avg)^e^**
1	1.40	0.00	0.54	−0.07	0.94	0.33
2	1.95	0.25	0.82	0.31	1.27	0.67
3	2.47	0.98	1.22	0.65	1.60	1.00
4	3.06	1.48	1.70	1.03	1.98	1.38
5	3.62	1.91	2.14	1.26	2.34	1.74
6	4.24	2.32	2.61	1.50	2.76	2.16
7	4.82	2.65	3.05	1.72	3.16	2.56
8	5.45	3.00	3.53	1.95	3.60	3.00
9	6.04	3.48	3.96	2.17	4.02	3.42

a All values with prime symbol denote cases in which log (D/T) values are estimated by taking the values provided in Table 2(C) part.

b In case of the first SOI value (1.40), the maximum (0.54) is taken from 4 values (−0.07, 0.31, 0.54, and 0.35) that are given in Table 2(C) part.

c In case of the first SOI value (1.40), the minimum (−0.07) is taken from 4 values (−0.07, 0.31, 0.54, and 0.35) that are given in Table 2(C) part.

d In case of the first SOI value (1.40), the sum is taken by putting the data in Table 2(C) part into the equation (=log (10^−0.07^ + 10^0.31^ +10^0.54^ + 10^0.35^) = 0.94).

e In case of the first SOI value (1.40), the average is taken by putting the data in Table 2(C) part into the equation (=log {(10^−0.07^ + 10^0.31^ +10^0.54^ + 10^0.35^) /4} = 0.33).
